# Plasma exchange in inflammatory demyelinating disorders of the central nervous system: reasonable use in the clinical practice

**DOI:** 10.1055/s-0042-1758447

**Published:** 2023-04-14

**Authors:** André Luiz Guimarães de Queiroz, Herval Ribeiro Soares Neto, Thiago Taya Kobayashi, Sonia Maria Cesar de Azevedo Silva

**Affiliations:** 1 Hospital do Servidor Público Estadual de São Paulo, Serviço de Neurologia, São Paulo SP, Brazil.

**Keywords:** Plasma Exchange, Demyelinating Autoimmune Diseases, CNS, Central Nervous System Diseases, Neuromyelitis Optica, Multiple Sclerosis, Troca Plasmática, Doenças Autoimunes Desmielinizantes do Sistema Nervoso Central, Doenças do Sistema Nervoso Central, Neuromielite Óptica, Esclerose Múltipla

## Abstract

Plasma exchange (PLEX) is a therapeutic apheresis modality in which the plasma is separated from inflammatory factors such as circulating autoreactive immunoglobulins, the complement system, and cytokines, and its therapeutic effect is based on the removal of these mediators of pathological processes. Plasma exchange is well established for various neurological disorders, and it is applied successfully in central nervous system inflammatory demyelinating diseases (CNS-IDD). It mainly modulates the humoral immune system; thus, it has a greater theoretical effect in diseases with prominent humoral mechanisms, such as neuromyelitis optica (NMO). However, it also has a proven therapeutic effect in multiple sclerosis (MS) attacks. Several studies have suggested that patients with severe attacks of CNS-IDD have poor response to steroid therapy but show clinical improvement after the PLEX treatment. Currently, PLEX is generally established only as a rescue therapy for steroid unresponsive relapses. However, there are still research gaps in the literature regarding plasma volume, number of sessions, and how early the apheresis treatment needs to started. Thus, in the present article, we summarize the clinical studies and meta-analyses, especially about MS and NMO, outlining clinical data regarding the experience with therapeutic PLEX in severe attacks of CNS-IDD, the clinical improvement rates, the prognostic factors of a favorable response, and highlighting the likely role of the early apheresis treatment. Further, we have gathered this evidence and suggested a protocol for the treatment of CNS-IDD with PLEX in the routine clinical practice.

## Resumo

Plasmaférese (PLEX) é um procedimento em que o plasma é separado de fatores inflamatórios como imunoglobulinas autorreativas circulantes, sistema complemento e citocinas, e seu efeito terapêutico se baseia na remoção desses mediadores de processos patológicos. A PLEX está bem estabelecida no tratamento de diversos distúrbios neurológicos, e é utilizada com sucesso em surtos de doenças desmielinizantes inflamatórias do sistema nervoso central (CNS-IDD). A PLEX modula principalmente o sistema imunológico humoral; assim, tem efeito teórico maior em doenças com mecanismos patológicos humorais proeminentes, como a neuromielite óptica (NMO). No entanto tem também efeito terapêutico comprovado em surtos de esclerose múltipla (EM). Estudos sugerem que a corticoterapia é pouco eficaz em pacientes com surtos graves de CNS-IDD, e que estes apresentam melhora clínica após o tratamento com PLEX. Atualmente, a PLEX está geralmente estabelecida apenas como terapia de resgate para surtos não responsivos a corticosteroides. No entanto, há lacunas na literatura sobre a quantidade de troca de volume plasmático, o número de sessões, e o tempo de início da aférese terapêutica. Dessa forma, resumimos neste artigo estudos clínicos e metanálises, especialmente sobre EM e NMO, e delineamos os dados clínicos sobre a experiência com o uso de PLEX em surtos graves de CNS-IDD, as taxas de melhora clínica, os fatores prognósticos para uma resposta favorável, e destacamos o provável papel do tratamento precoce nestes casos. Em um segundo momento, reunimos essas evidências em uma sugestão de protocolo de tratamento de CNS-IDD com PLEX na prática clínica rotineira.

## INTRODUCTION

Plasmapheresis stems from the Greek term *apheresis* , which means to “take away by force” or “withdraw”. Plasmapheresis, or plasma exchange (PLEX), is the filtration of plasma, in which the goal is to remove a given volume of the patient's plasma, separate it from corpuscular blood constituents, and replace the plasma with a substitute fluid, reinfusing it to the patient. 1 The therapeutic effect of apheresis procedures aims to remove deleterious targeted components contained in the plasma. It typically involves the elimination of pathogenic and autoreactive immunoglobulins, the complement system, and cytokines; thus, it is mainly based on a modulation of the humoral immune system.

Removal of humoral factors might also modulate cellular components of the immune system. Linkage occurs especially through cell types with receptors for immunoglobulins, such as natural killer cells, monocytes, and macrophages. Yet, such effects on cellular immune responses have not been completely understood to date. Recent findings 2 3 show that extracorporeal removal of immunoglobulins might also impact on immune responses mediated by T cells in addition to the immunoglobulin reducing effect.

Today, the PLEX therapy is the standard of care for a diverse array of diseases. It is currently well established to treat a number of acute neurological conditions, including central nervous system inflammatory demyelinating diseases (CNS-IDD). The first line of treatment for acute relapses of CNS-IDD, mostly represented by multiple sclerosis (MS) and neuromyelitis optica spectrum disorders (NMOSD), is high doses of corticosteroids. In severe acute attacks, however, steroids are insufficient because of their poor responses, 4 5 which account for about 5% of cases of CNS-IDD. 6 The clinical symptoms that represent the severity criteria are summarized in Table 1 . There is evidence in the literature that PLEX is effective in about 45% of steroid-refractory cases, with sustained benefit. 7 In this setting, the molecular basis of PLEX is the local deposition of antibodies and complement in acute demyelinating diseases, as determined by histopathological studies. 2 8

**Table 1:** Severity criteria for demyelinating-inflammatory attacks of the central nervous system

Optic neuritis
1. Bilateral optic neuritis
2. Severe visual loss (visual acuity < 20/200)
Brain injury
1. Hypothalamic lesion
2. Bulbar lesion
Acute myelitis
1. Symptoms compatible with transverse/complete myelitis
2. Major motor deficit
3. Bladder dysfunction

There are still research gaps, with only theoretical considerations on the frequency, number of sessions, and how early the apheresis treatment needs to be started. The aim and motivation for the present review is to summarize the scientific evidence regarding the experience with therapeutic PLEX in severe attacks of CNS-IDD, especially MS and NMOSD, as well as the rate of improvement and the prognostic factors for a favorable response. In a second moment, we summarize this evidence and suggest a protocol for the treatment of CNS-IDD with PLEX in the routine clinical practice.

## PLASMA EXCHANGE IN MULTIPLE SCLEROSIS

Multiple sclerosis relapses are usually associated with significant functional impairment, and, in the case of incomplete remissions, are an important contributor for neurologic disability and decreased health-related quality of life. Therefore, a fast and aggressive treatment is critical.

The initial recommendation for the management of acute MS relapse is the administration of intravenous methylprednisolone (IVMP), 1,000 mg/day over a period of 3 to 5 consecutive days. 9 It is believed that corticotherapy can decrease edema, due to its antiinflammatory component, reduce the number of B-lymphocytes and their availability at the inflammatory sites in the CNS, and improve the blood–brain barrier permeability dysfunction, resulting in fewer gadolinium-enhancing lesions. 9 10

Nevertheless, about one quarter of relapses present insufficient improvement after IVMP administration, 11 and, in such cases, several options, such as PLEX, have been studied and used. Due to the available evidence, European and American guidelines consider that patients with MS who have not responded to the treatment with IVMP may benefit from PLEX as a second-line therapy. 12 13 The Brazilian Consensus for the Treatment of Multiple Sclerosis also considers the use of PLEX in patients who fail to respond or have contraindications to corticosteroids. 14

The sustained efficacy of PLEX in patients with steroid-refractory MS relapses has been consistently supported in several studies, 3 11 15 16 17 18 as summarized in Table 2 . In 1999, Weinshenker et al. 15 first demonstrated the benefit of PLEX in a randomized, sham-controlled, double-blinded trial with patients with an acute severe neurological deficit caused by MS and other demyelinating diseases of the CNS. Regarding the patients who did not achieve a marked or moderate improvement with high-dose glucocorticoids within the last 3 months of the treatment, 42% presented relevant improvement with PLEX, compared with only 6% after the sham treatment. In a 2009 study by Trebst et al., 18 18 out of 20 patients with acute MS relapses presented marked-to-moderate improvement after therapeutic PLEX. These findings in these two prospective studies 15 18 were corroborated by several retrospective trials as well as small case series, with an improvement in at least 60.9% 19 of MS patients or regard clinically isolated syndrome. 6 17 18 19 20 21 22 In more recent studies, Blechinger et al. 22 reported a rate of 78.8% of clinical improvement in 118 MS patients, and Pfeuffer et al. 19 reported a rate of 60.9% of good/full recovery following treatment with PLEX, and of 6.5% of no or worse recovery in 6.5% PLEX-treated patients against 69.7% of IVMP-treated patients ( *p* < 0.001). A Portuguese cohort 17 study found a rate of 41.3% of complete recovery of neurological disability and of 39.1% of partial recovery; moreover, a higher number of PLEX sessions was associated with better clinical recovery, endorsing the need to adjust the number of apheresis courses to the specific clinical situation. Notably, a recent systematic review by Rolfes et al. 11 highlighted that the timing of the start of the apheresis is a strong predictor of a good outcome, which will be herein discussed in depth later. The association with beneficial PLEX response considers also younger age, the male sex, shorter MS disease duration, and a relapse not manifesting as optic neuritis (ON). 11

**Table 2:** Overview of publications on therapeutic plasma exchange in acute multiple sclerosis relapse

Author	Year	Journal	Study design	Number of MS patients	Relevant results	Ref.
Weinshenker et al.	1999	*Annals of Neurology*	Prospective, randomized	12	Marked to moderate improvement in 42.1% of CNS-IDD patients after PLEX versus. 5.9% after sham.	15
Keegan et al.	2002	*Neurology*	Retrospective	22	Marked to moderate improvement in 41% of the patients. Early initiation of therapy was associated with greater improvement.	16
Keegan et al.	2005	*Lancet*	Retrospective	19	MS patients with pattern-II pathology are more likely to respond favorably to PLEX than patients with patterns I or III.	23
Llufriu et al.	2009	*Neurology*	Retrospective	23	Improvement in 52% MS patients in 6 months of follow-up. Early initiation of PLEX was a predictor of a good response.	3
Trebst et al.	2009	*Blood Purification*	Prospective	20	Marked to moderate improvement in 76% of patients with optic neuritis and in 87.5% of patients with relapse other than optic neuritis.	18
Habek et al.	2010	*Therapeutic Apheresis and Dialysis*	Retrospective	4	Marked to moderate improvement in 75% of the patients.	21
Magaña et al.	2011	*Archives of Neurology*	Retrospective	55	Marked to moderate improvement in 62% of the patients. Shorter disease course was associated with a favorable PLEX outcome.	6
Brunot et al.	2011	*Presse Medicale*	Retrospective	15	Early initiation of PLEX was a predictor of a good response.	45
Ehler et al.	2014	*Therapeutic Apheresis and Dialysis*	Retrospective	11	Improvement in 72.7% of IVMP-unresponsive CIS patients after PLEX.	20
Correia et al.	2018	*Multiple Sclerosis and Related Disorders*	Retrospective	46	Improvement with complete recovery according to the EDSS in 41.3% of the patients and partial in 39.1%. The subgroup of patients who experienced complete recovery according to the EDSS had the highest number of PLEX sessions.	17
Stork et al.	2018	*JAMA Neurology*	Retrospective	69	Both patterns I and II improved clinically after the PLEX treatment, but pattern II benefited the most.	24
Pfeuffer et al.	2019	*Journal of Clinical Medicine*	Retrospective	66	Good recovery at discharge was observed in 60.9% of the PLEX patients versus 15.2% of the IVMP patients.	19
Palacios-Mendoza et al.	2020	*Neurological Sciences*	Retrospective	32	Improvement in 59.4% of the patients at discharge and in 60.9% after 6 months. Early improvement with PLEX was a predictor of good response at 6 months of follow-up.	38
Blechinger S.	2021	*Therapeutic Advances in Neurological Disorders*	Retrospective	118	Marked to moderate improvement in 78.8% of the patients. Early initiation of PLEX was a predictor of a good response.	22

Abbreviations: CID, Clinically Isolated Syndrome; CNS-IDD, central nervous system inflammatory demyelinating diseases; EDSS, Expanded Disability Status Scale; IVMP, intravenous methylprednisolone; MS, multiple sclerosis; PLEX, plasma exchange; Ref., reference.

There is much evidence that implicates B cells, plasma cells, antibodies and complement in the MS pathology; thus, treatments to deplete them, such as PLEX, are indeed effective. However, sources point out that the response to PLEX seems to be variable based on the immunopathological heterogeneity in MS. 7 16 Some patients seem to respond promptly to PLEX, while others do not. To explain this, some authors propose that factors such as MS predominance of different immunopathologies among patients could contribute to the variations in the response to PLEX. 23 Based on a large series of diagnostic brain biopsies from MS patients, a group of three international centers coined a four-pattern classification of actively demyelinating MS lesions according to immunopathological characteristics with distinct mechanisms of demyelination. 8 These four immunopathological patterns have been described as:

- Pattern I: defined by the occurrence of T cell/macrophage-associated demyelination;
- Pattern II: characterized by immunoglobulin deposition and complement activation with antibody/complement-associated demyelination;
- Pattern III: defined by distal oligodendrogliopathy; and
- Pattern IV: characterized by oligodendrocyte degeneration in periplaque white matter.

To support this classification, in one study 23 that investigated the response to PLEX according to immunopathological patterns in patients who had undergone brain biopsy, all patients with pattern-II pathology experienced moderate to marked improvement compared to none of the remaining patients with different patterns, leading to a hypothesis that patients with other subtypes of MS lesions could not respond to PLEX. A recent single-center cohort study 24 with 69 MS patients with pathologies of patterns I, II, and III showed improvement after PLEX among patients with patterns I and II, but the pattern-II patients benefited the most. Patients with pattern-III lesions did not benefit from PLEX. This selective response reaffirms that the mechanism of action of PLEX relies on the removal of immunoglobulins, complement factors, cytokines, and immune complexes, and probably not as much on T cell and macrophage inflammation, which is similar across all other three immunopathological patterns except pattern II. 23 The evidence presented suggests that the response to the apheresis treatment is associated with the immunopathological pattern, indicating that the pattern with the most humoral immune response benefited the most. This concept of immunopathologically different MS subtypes provides a strong point for individualized therapeutic approaches.

Considering the aforementioned information, we agree that PLEX should be considered in the case of relapse without complete recovery after IVMP as an escalation therapy for steroid-unresponsive MS relapses. The decision to perform PLEX should be made on an individual basis, with an early clinical assessment of the severity of the MS relapse and in case of poor or no response to the corticosteroid therapy. The identification of local deposits of humoral factors by brain biopsy to diagnose a specific pathologic subtype is usually unavailable in the clinical practice, and it does not yield enough benefits to be routinely performed in MS patients.

Based on all observations and results discussed above, it's confirmed the support of use of PLEX in patients with acute severe attacks who fail to improve after high-dose corticosteroids treatment. A shorter delay after steroid failure could be advocated to maximize the chances of improvement with PLEX. 11 There is no consensual protocol, but no less than five PLEX courses should be administered on alternate days to achieve a rapid biological effect. Fulminant attacks are treated more aggressively, with the possibility of extending the PLEX sessions. The apheresis procedure is effective and relatively safe for most patients.

## PLASMA EXCHANGE IN NEUROMYELITIS OPTICA SPECTRUM DISORDERS

It was previously thought that Neuromyelitis optica (NMO) was an aggressive variant of MS, but nowadays is a better understood autoimmune demyelinating disease of the CNS. NMO demyelinating lesions are characterized by deposition of complement, immunoglobulins and neutrophils and eosinophils infiltration; 4 these lesions correspond to pattern II of the aforementioned classification, and they improve with PLEX. 8 25 In 2004, a specific antibody, called aquaporin-4-immunoglobulin G (AQP4-IgG), was first described as a new biomarker associated with NMO. 26 In recent years, studies have demonstrated that astrocytes were selectively targeted in NMO, as AQP4-IgG is involved in a complement dependent toxicity against the astrocytes. 4 These findings are currently recognized as the main pathogenic factors in the pathophysiology of the disease. 2 With the PLEX treatment, the anti-AQP4 antibodies can be reduced to less than 20% of their initial level. 1 4 27 IgG, IgM and complement are excluded from the circulating plasma and cannot migrate anymore to the lesions, suppressing active CNS inflammatory attack.

In summary, NMO attacks are then characterized by AQP4-IgG antibody directed against water channel protein AQP4, activating the antibody/complement system, cascade and leading to the destruction of astrocytes and neuronal tissue, in which a wide range of demyelinating lesions could occur, including large necrosis. 4 Therefore, NMO relapses are usually more severe than MS relapses, and most NMO patients often present accumulated disability due to poor recovery from the attacks, resulting in a worse prognosis for this disorder. Although immunosuppressive drugs prevent part of NMO relapses, successful and timely treatment of acute attacks is decisive for the long-term outcome, reducing residual deficits and disability. 6 25 28

High-dose IVMP generally constitutes the first-line treatment for NMO relapses. As aforementioned, the widely-used steroid treatment usually fails to control severe attacks of CNS-IDD. 4 Also, NMO may be less responsive to steroids compared to other types of CNS-IDD, 4 5 for an expressive proportion of NMO patients are often found to be unresponsive to steroids during acute attacks. 23 29 30 In a large NMO study, 29 693 attacks in 181 patients were treated with high-dose steroids as the first-line therapy. Out of this cohort, 17% recovered completely, 65.4% were partial responders, and 16.2% did not respond at all. Therapy escalation with PLEX was markedly effective, particularly in decreasing the proportion of steroid non-responders. A recent meta-analysis of 24 studies 31 showed a reduction in the mean score on the Expanded Disability Status Scale (EDSS) of about 2 points in NMO patients and an efficacy regarding the rate of response to PLEX of 74%, supporting the protective role of PLEX in this disorder. Accordingly, the American Society for Apheresis (ASFA) recommends therapeutic PLEX as a second-line therapy, either as stand-alone treatment or in conjunction with other modes of treatment, which is category II indication, with grade 1B recommendation (strong recommendation, moderate quality evidence), in the treatment of acute NMO attacks. 32

As aforementioned, the first prospective, randomized, double-blinded study on CNS-IDD was carried out in 1999 by Weinshenker et al., 15 and it included NMO patients who failed to recover after treatment with high-dose IVMP but subsequently experienced marked therapeutic benefits after PLEX. Since then, retrospective and prospective studies as well as case series have reported significant improvement in around 42 to 82.8% of NMOSD patients treated with PLEX 6 16 27 33 34 35 36 37 38 39 (for an overview, see Table 3 ). Functional improvement occurs regardless of the AQP4-IgG serostatus. 5 6 36 Some studies even suggest starting PLEX as a first-line therapy in an acute attack. In 2021, a meta-analysis 31 of 4 studies involving 80 patients evaluated the efficacy of PLEX as a first-line therapy, with a rate of response of 71% (95% confidence interval [95% CI]: 44–93%). In patients with high EDSS scores, Kumawat et al. 36 demonstrated a very favorable outcome with prompt PLEX initiation, and they also proposed apheresis as a first-line treatment. Bonnan et al. 40 reported great improvement with the reduction in the delay in initiating the PLEX treatment to preferably ≤ 5 days, warning against PLEX only as a rescue therapy after steroid failure.

**Table 3:** Overview of publications on therapeutic plasma exchange in acute neuromyelitis optica relapse

Author	Year	Journal	Study design	NMO patients	Relevant results	Ref.
Keegan et al.	2002	*Neurology*	Retrospective	10	Marked to moderate improvement in 60% of the patients. Early initiation of therapy was associated with greater improvement.	16
Watanabe et al.	2007	*Multiple Sclerosis Journal*	Retrospective	6	Moderate improvement in 50% of the patients unresponsive to IVMP. The clinical improvement started to appear after one or two exchanges.	34
Bonnan et al.	2009	*Multiple Sclerosis Journal*	Retrospective	34	Residual EDSS and lowering of EDSS scores were significantly better in the PLEX-treated group than in the group treated only with IVMP.	5
Llufriu et al.	2009	*Neurology*	Retrospective	4	Improvement in 75% NMOSD patients in 6 months. Early initiation of PLEX was a predictor of a good response.	3
Magaña et al.	2011	*Archives of Neurology*	Retrospective	26	Marked to moderate improvement in 42% of the patients. A shorter disease course was associated with a favorable PLEX outcome.	6
Kim et al.	2013	*Journal of Clinical Neurology*	Retrospective	15	PLEX following IVMP therapy led to significant improvement in 50% of the attacks after the procedure and in 78% after 6 months.	27
Kleiter et al.	2016	*Annals of Neurology*	Retrospective	186	First-line therapy with PLEX was superior to IVMP in attacks involving the spinal cord.	29
Abboud et al.	2016	*Multiple Sclerosis Journal*	Retrospective	59	65% of patients using IVMP concurrently with PLEX achieved an EDSS score ≤ their baseline against 35% in IVMP-only patients.	35
Aungsumart and Apiwattanakul .	2017	*Multiple Sclerosis and Related Disorders*	Retrospective	24	PLEX following IVMP therapy led to a significant improvement in 81% of the cases after 6 months of follow-up.	39
Kleiter et al.	2018	*Neurology: Neuroimmunology & NeuroInflammation*	Retrospective	105	A strong predictor of complete remission was the use of PLEX as a first-line therapy. Immediate start within 2 days of symptom onset was associated with greater degree of recovery.	47
Jiao et al.	2018	*Clinical Therapeutics*	Retrospective	29	Improvement in 82.8% of the patients at 1 month after PLEX. Early PLEX initiation was an independent prognostic factor.	33
Kumar et al.	2018	*Annals of Indian Academy of Neurology*	Retrospective	5	Marked to moderate improvement in 60% of severely disabled IVMP-refractory patients.	37
Bonnan et al.	2018	*Journal of Neurology, Neurosurgery, and Psychiatry*	Retrospective	55	Early initiation of PLEX (≤ 5 days) was more beneficial than delayed PLEX. The study suggests a better outcome if PLEX is started before day 2 of the relapse.	40
Srisupa-Olan et al.	2018	*Multiple Sclerosis and Related Disorders*	Retrospective	52	IVMP non-responders but PLEX responders showed continuous and maximum improvement at 6 months follow-up. Patients who received PLEX had a significantly lower relapse rate compared with those who received IVMP alone.	42
Kumawat et al.	2019	*Annals of Indian Academy of Neurology*	Retrospective	30	Improvement in 73.3% of the patients following PLEX. The only predictor of a good outcome was early initiation of therapy.	36
Songthammawat et al.	2020	*Multiple Sclerosis and Related Disorders*	Prospective, randomized	11	A trend towards a better outcome with early PLEX initiation and with IVMP and PLEX combined.	46
Palacios-Mendoza et al.	2020	*Neurological Sciences*	Retrospective	15	Improvement in 46.7% of the patients at discharge and in 70% after 6 months.	38
Kosiyakul et al.	2020	*Annals of Clinical and Transnational Neurology*	Meta-analysis	241	All studies consistently demonstrated the benefit of PLEX with improved visual acuity and decreasing EDSS scores.	41
Huang et al.	2021	*Multiple Sclerosis and Related Disorders*	Meta-analysis	228	The initiation time of PLEX significantly reduced the EDSS score in NMO patients and the optimal timing for PLEX was 8 to 23 days after the onset of the disease.	44
Yu et al.	2020	*Journal of Neuroimmunology*	Meta-analysis	528	PLEX treatment as a recue therapy resulted in a reduction in the mean EDSS score of 1.7, with a response rate of 75%. As a first-line therapy, PLEX resulted in a reduction in the mean EDSS score of 2.3, with a response rate of 71%.	31

Abbreviations: EDSS, Expanded Disability Status Scale; IVMP, intravenous methylprednisolone; MS, multiple sclerosis; NMO, neuromyelitis optica; NMOSD, neuromyelitis optica spectrum disorders; PLEX, plasma exchange; Ref., reference.

In the early stages of acute lesions, a transient phenomenon occurs, in which the AQP4 protein targeted by the complement system and autoantibodies is internalized and downregulated. 25 What seems to happen according to in vitro experiments is that internalized AQP4 is initially protected from necrosis, and the lesion grows a necrotic core surrounded by a “penumbra”. 40 Consequently, irreversible necrotic lesions could be prevented through an early, aggressive treatment. 4 There is a growing body of evidence for better prognostic outcomes with an earlier performance of PLEX in acute attacks, 3 as we will address herein later.

There is also evidence of long-term benefits after PLEX. A 2020 meta-analysis 41 found that treatment with PLEX during an acute attack is also associated with a significantly decreased EDSS score after 6 months and 1 year of follow-up, with a mean difference of 2 points comparing pretreatment to posttreatment. The depletion of B cells explains to a certain extent the prolonged benefit after apheresis. The AQP4-IgG antibody is produced by the differentiation of B cells into plasma cells. The reduction of B cells by PLEX avoids the reexpansion of autoreactive B cells, thus helping to reduce the production of AQP4-IgG in the long term, similar to other B-cell depletion therapies. In a Thai study, 42 steroid-refractory NMO patients who had undergone PLEX experienced larger and more sustained improvements at the 6-month follow-up in comparison to steroid-responsive NMO patients.

A study by Ito 43 has proposed that the autoimmune-mediated disruption of the AQP4 water channel function may predispose to posterior reversible encephalopathy syndrome in NMO patients and seems to increase the risk in patients who experience fluctuations in blood pressure or who are treated with therapies that can cause fluid changes, as occurs in aphereses.

In conclusion, the usual management followed by experts in NMO attacks is that every relapse needs to be treated fast. High-dose IVMP is a good start agent, mainly because it is widely available, simple to administer, and it may provide some benefits in suppressing the acute inflammatory response, but even if high-dose steroids reduce the inflammatory cellular response, they are clearly not sufficient in some cases, especially in clinically-severe inflammatory attacks. Poor outcomes are still a common issue in NMO, even when the steroid treatment is administered immediately after onset. 4 As a therapeutic option, PLEX is of critical importance, and it is beneficial in acute NMO attacks whether used as a rescue therapy or as a first-line therapy, and long-term therapeutic effects can also be noted.

## THE TIME BETWEEN THE ONSET OF AN ATTACK AND THE INITIATION OF TREATMENT IS KEY

In multiple studies, treatment effectiveness was associated with the time from disease onset to the initiation of apheresis. In a meta-analysis by Huang et al, 44 the initiation time of PLEX significantly affected the outcomes (reduction in the EDSS score) of NMO patients, and optimal timing for PLEX was of 8 to 23 days after the onset of the disease. In another two studies 3 16 with CNS-IDD patients, the highest response rates were observed when PLEX was started before 16 days 3 and 21 days. 16

A shorter PLEX delay was defined as a predictor of a good outcome in multiple studies, with strong evidence in several CNS-IDD series. 3 16 18 33 36 37 40 45 46 47 Early initiation of PLEX should be considered in the clinical practice, given that it is a variable that can be controlled to a certain extent if there is access to treatment.

Moreover, some authors have observed that apheresis could be advantageous even as the first treatment course for attacks affecting the CNS, shortening the duration between disease onset and PLEX as much as possible, proposing a new and decisive sequence of therapies. 27 29 36 37 40 46 47 Bonnan et al. 40 observed a few convincing cases of Lazarus effect (immediate return to baseline clinical line after a severe attack) in patients treated on the first day after a demyelinating attack. The probability of experiencing complete improvement in this study 40 continuously decreased as the delay in receiving PLEX increased, from 50% at days 0 to 1 to approximately 5% after day 20 ( *p* = 0.02). After adjustment by multivariable logistic regression, the probability of experiencing complete improvement was associated with PLEX delay ( *p* = 0.01). 40

It is important to take into account that it takes time for patients to transfer from primary medical care to a tertiary referral hospital—especially in developing countries—, not to mention the additional time to assess whether the patient responds to IVMP. Therefore, Huang et al. 44 indicate that it is reasonable to set the PLEX initiation time window between 8 and 23 days.

The superior clinical improvement with the early performance of PLEX is likely because prolonged inflammation can cause more severe demyelination and, therefore, worse axonal damage. 16 One requirement for better recovery of the neurologic function is the survival of the affected nerves. However, this does not mean that PLEX has no therapeutic effect if delayed. It is important to note that some studies have shown patients experiencing recovery from their disability even if the apheresis is performed long after the relapse. 48 Correia et al. 17 found no significant differences in MS patients who underwent PLEX during the first month after relapse onset and after 3 months, although they observed a tendency of more partial/complete disability recovery in the 1st month (83%) than in the 3rd month (75%). In a study by Keegan et al., 16 patients treated after 60 days often experienced a favorable response, and the authors state that they should not be excluded from treatment if the onset of the neurologic event is not exactly recent

Although there is much evidence on the benefit of shorter delays in performing PLEX as mentioned, some studies have failed to demonstrate an impact of early PLEX initiation on outcomes. 6 17 38 49 Nevertheless, it is important to take into account again that the time of PLEX initiation is a controllable variable, and has no downside (except for the cost and treatment access in different hospitals), because it will delay IVMP treatment.

Especially regarding NMOSD, there is stronger evidence that the earlier initiation of PLEX results in more favorable outcomes. Bonnan et al. 40 showed that initiating PLEX within 5 days is a strong predictor of complete remission of severe NMO attacks. In a retrospective study of 207 therapeutic interventions through apheresis by Kleiter et al., 47 an immediate start within 2 days of symptom onset was associated with a greater degree of recovery even when compared with delays of only 1 week. Given the prior information, it may be harmful to perform PLEX only after steroid failure, as a rescue therapy, especially in patients with severe clinic presentation. High-dose steroid infusion may take 3 to 5 days to show potentials of improvement, and this delay is added to others, like those experienced between symptom onset and hospital admission and between admission and treatment initiation.

As shown, incorporating PLEX earlier or even as part of the first-line therapy in CNS-IDD, especially on cases of NMO relapse, should be considered, given the overall poorer prognosis associated with such attacks. From a practical point of view, we recommend considering PLEX in every severe attack of an inflammatory demyelinating disorder. The PLEX treatment should be started as soon as possible, in addition to steroids. Although less than 1% of the circulating steroids are removed by PLEX, when used concomitantly, steroids should be preferably infused at the end of each PLEX session.

## FINAL REMARKS

Plasmapheresis, despite being an invasive treatment method that has an impact on hemodynamic physiology, has been shown to be an alternative therapeutic procedure with proved clinical benefits for patients with CNS-IDD attacks. These benefits are crucial because these disorders result in important functional impacts, such as limitations to ambulation, vision, and sphincter control. The PLEX treatment has been proven to be effective in CNS-IDD patients, with a favorable profile on terms of adverse effects. It is well tolerated and has a low incidence of serious complications. The rate of complications varies due to multiple factors, including the experience of the health care center, and the age and preexisting comorbidities of the patients.

Several studies have highlighted possible predictors of good response after PLEX. 3 6 16 38 39 40 Some predictors were not always found in every study, such as the male sex, retained reflexes, 16 gadolinium-enhancing lesions on magnetic resonance imaging (MRI), and short disease duration, 39 and some predictors were a consistent finding in different studies, such as the early initiation of PLEX, as aforementioned. 3 4 16 27 29 33 36 40 44 45

Although initiating PLEX as early as possible seems effective, especially in the treatment of acute NMO relapses, from a practical and realistic point of view, it may be difficult to bypass steroids as the first-line therapy, especially in underdeveloped countries.

Side effects, costs, and a shared decision with the patient must be taken into account when choosing a treatment. Combining IVMP with PLEX seems to be the best option to manage severe CNS-IDD relapses in the clinical practice. The time to start PLEX should be determined by the attending physician depending on the severity of the relapse and the clinical condition of the patient.

The PLEX treatment has been proven to be always appropriate in NMO attacks, but MS relapses have been predicted to respond more properly to PLEX if they mostly present an immunopathological pattern of immunoglobulin/complement system deposition (type-2 lesions). As a practical matter, in cases of MS relapse, PLEX should be used as add-on treatment in extremely severe attacks or as soon as steroid failure is noted. No factor during an acute relapse has been robustly demonstrated to be a predictive risk of steroid failure to the point that it could be used to select high-risk patients to receive PLEX immediately. Therefore, PLEX should be considered at the end of every steroid infusion, and the clinical response should be evaluated on a daily basis. The clinical severity of the relapse at its onset is the ultimate criteria that should base the decision of the clinician to perform PLEX as soon as possible.

Regarding the volume of plasma and the number of apheresis courses, all standard recommendations are based on theoretical considerations, practical observations, and issues pertaining to risks and costs. 1 The current recommendation is of 5 to 7 sessions to substantially reduce the levels of autoantibodies, with a variance in use in the literature of 5 to 15 sessions. However, the exact number of exchanges with statistical evidence for a better clinical prognosis remains to be determined.

According to some studies, exchange molecules get reduced to less than 20% of their initial levels after 5 exchanges. 1 Thus, a minimum of 5 sessions are necessary to effectively remove the IgG antibodies, such as AQP4-IgG, in NMO attacks. 27 Similarly, previous studies have suggested that five or six standard plasmapheresis sessions are required to substantially reduce blood levels of IgG. 27 In general, if the resynthesis of autoantibodies and inflammatory substances is slow, at least 5 sessions of PLEX in a period of 7 to 10 days are necessary to remove 90% of the initial inflammatory components. However, it is necessary to take into account that, if the rate of production of inflammatory components is high (autoantibodies, components of the complement system), additional sessions are required, and they have to be titulated according to clinical evaluation. 50

## SUGGESTION OF TREATMENT PROTOCOL

In addition to providing a background overview for clinicians and neurologists about PLEX and acute demyelinating relapses, the present article aims to present a guide for therapeutic decisions in these severe inflammatory attacks. This protocol was created for our institution based on the present literature review and the opinions of specialists. The main aim for the protocol is to prevent increases in disability and achieve the greatest clinical improvement possible in patients with acute deficits in these clinical settings.

**Figure 1:**
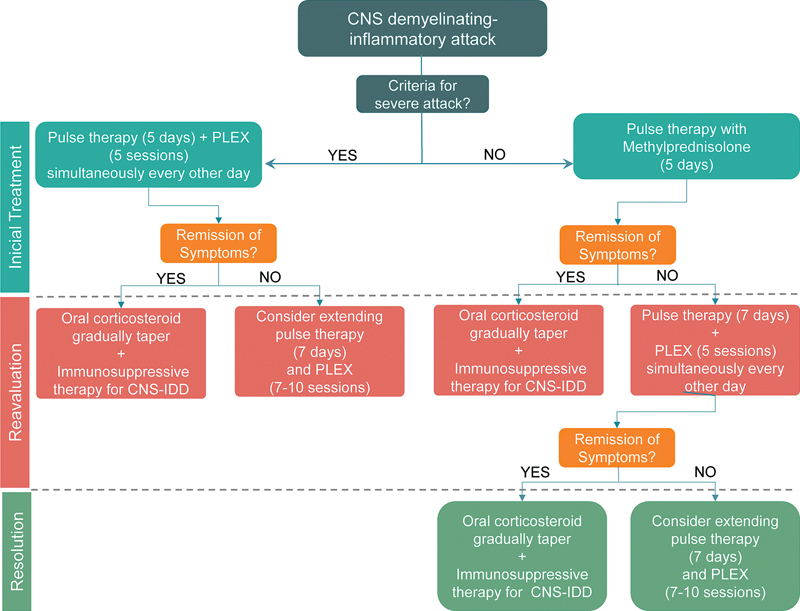
Suggestion of a protocol for the treatment of central nervous system inflammatory demyelinating diseases with plasma exchange in the routine clinical practice.

Well-designed, prospective clinical trials are needed to evaluate the real clinical benefits of PLEX in different types of demyelinating disorders. However, until the results of these future trials are known, it is our hope that the standardized PLEX protocol and assessment measures suggested in the present document may be useful for clinicians who are considering trying PLEX as a treatment option. With the aforementioned consistent flow of evidence, we suggest an algorithm ( Figure 1 ) for the therapeutic conduct with PLEX in acute outbreaks of CNS-IDD.

Firstly, we recommend the identification of the signs of severity of these attacks: visual acuity lower than 20/200 and bilateral impairment (optic neuritis), hypothalamic or bulbar lesions (brain injuries), and signs of transverse/complete myelitis, major motor deficit, and/or bladder dysfunction (acute myelitis). We suggest, as the first-line treatment, the performance of pulse therapy with methylprednisolone, 5 g in 5 days. In case of signs of clinical severity, consider incorporating PLEX as an adjunct to the first-line therapy, alternating with corticosteroid therapy. If the patient does not show clinical improvement in the daily clinical assessment, we suggest extending the corticosteroid pulse therapy to 7 g in 7 days 51 52 concurrently with PLEX in 7 to 10 courses. Some authors go further and extend IVMP regimens that range from 3 g infused in 3 days to 10 g infused in 10 days 4 in selected cases.

In conclusion, the present paper attempts to provide an overview of the general principles and scientific data that should guide therapeutic decisions in severe demyelinating attacks. We highlight the inclination to start PLEX early in the course of severe outbreaks, not always waiting for failure of the IVMP treatment to prevent the increase in disability. We advocate flexibility in PLEX management, tailoring the number of sessions, the time of initiation, and the concomitant IVMP treatment to individual circumstances, especially the clinical setting and the severity of the attack.

We acknowledge that these recommendations do not cover all situations that neurologists and clinicians may face in the management of CNS-IDD. It is good practice for the treating physician to always review up-to-date information, and to check all relevant clinical information and evaluate it on an individual basis. Regular revisions of guidelines are necessary, since the understanding about diseases increases and new treatments become available.
